# Challenges for malaria surveillance during the COVID-19 emergency response in Nampula, Mozambique, January - May 2020

**DOI:** 10.11604/pamj.2021.38.254.27481

**Published:** 2021-03-11

**Authors:** Gerson Afai, Auria Ribeiro Banze, Baltazar Candrinho, Cynthia Sema Baltazar, Erika Valeska Rossetto

**Affiliations:** 1 Field Epidemiology and Laboratory Training Program, National Institute of Health, Maputo, Mozambique,; 2 National Institute of Health, Maputo, Mozambique,; 3 National Malaria Control Program, Ministry of Health, Maputo, Mozambique,; 4 MassGenics assigned to Mozambique Centers for Disease Control and Prevention, Maputo, Mozambique

**Keywords:** Malaria, COVID-19, health impact assessment, Mozambique

## Abstract

Since the announcement of the coronavirus disease (COVID-19) pandemic in January 30^th^ 2020, 68 countries reported to the World Health Organization that they were experiencing disruptions in malaria diagnosis and treatment. This situation had the potential to lead to delays in diagnosis and treatment, which could result in an increase in severe cases and deaths. This analysis was based on findings from a field visit, carried out between June 30^th^ and July 1^st^, 2020, to a warehouse, to two health facilities, and a meeting with a community health worker, and an descriptive epidemiologic data analysis of health information system (HIS) to evaluate trends of the number of people tested for malaria and number of malaria cases reported, by comparing data from 2018, 2019 and 2020 for the period between January and May. The two health facilities and the warehouse had about two months of stock of antimalarial drugs, and patients with malaria symptoms were being tested for malaria at the COVID-19 screening site. The HIS data showed that the number of reported malaria cases decreased by 3.0% (177.646/172.246) in April, and 7.0% (173.188/161.812) in May, when comparing 2019 and 2020 data. People tested for malaria in community increased by 39.0% (190.370/264.730), between 2019 and 2020. The COVID-19 may have had a negative impact on the diagnosis and treatment of malaria in health facility (HF). The decrease in people tested for malaria in the health facilities may have overwhelmed the activities of the community.

## Introduction

By October 11^th^ 2020, over 37 million of 2019 coronavirus disease (COVID-19) cases and 1 million deaths were reported worldwide [1]. In the same period, the number of COVID-19 cases in Mozambique was 10.001, with 71 deaths and Nampula province with 497 reported cases, and five deaths. On June 6^th^, Nampula Province was declared the first Mozambican province with COVID-19 community transmission [2,3].

At a time that the world is plagued by COVID-19, challenges are immense in sub-Saharan Africa malaria-endemic countries to ensure the continued functionality of malaria prevention interventions, such as the distribution of insecticide-treated mosquito nets, access to effective diagnosis and treatment. At the same scenario, 46.0% of the 68 countries reported to World Health Organization (WHO) an experiencing disruption in malaria diagnosis and treatment, and Mozambique is not part of these countries [4,5]. This study aimed to evaluate the impact of COVID-19 on malaria surveillance activities, through a site visit conducted in two districts of Nampula province to identify the main challenges for malaria control and its trends during the state of emergency under COVID-19 in Mozambique.

## Methods

**Study design and setting:** the first step of this study was to conduct a site visit during June 30 and July 1^st^, 2020, to the warehouse and one health facility (HF) in Nampula City, and one HF and a meeting with community health worker (CHW) in Ribaue District. We tried to find out about the flow of screening for febrile patients who requested medical care in the two HF. The second step was conducting a cross-sectional descriptive epidemiological data analysis, to evaluate the trend of people tested and malaria cases from January to May of the years 2018, 2019, and 2020 in Nampula Province.

**Data collection:** the data collected during the site visit was through an interview and verification of drug stock logbooks to verify the availability of antimalarial drugs and malaria rapid diagnose test (RDT) for the next months (August and September, 2020). The malaria data for epidemiological data analysis were from the health information system (HIS), and were provided by the Nampula Provincial Health Directorate in an electronic spreadsheet. The case definition of malaria case was considered a patient presenting with signs and symptoms suggesting malaria with positive microscopy or RDT result.

**Data analysis:** a descriptive analysis was performed using Microsoft Excel for Mac version 16.44. The trend of people tested and malaria cases was through the calculation of percentage evolution, and graphical representation from January to May of the years 2018, 2019, and 2020.

**Ethical considerations:** this study was carried out in the technical support framework of malaria surveillance, in the ambit of the COVID-19 emergency, ethical approvement was not required, and the malaria data analyzed were grouped and did not involve Individual identification.

## Results

### Malaria diagnosis and treatment supplies

In the warehouse of Nampula City, it was found that the artemether-lumefantrine (AL) was the most consumed antimalarial drug, and the stock of sulfadoxine-pyrimethamine (SP), injectable quinine were to cover about a month and a half, while the stock of RDT was to cover about two months at most. The artesunate suppository, for rectal use, was supplied through the medicine kits for use by the CHW. In both visited HF, the stock of SP, injectable quinine, and RDT was to cover approximately two months at most.

### Malaria screening

In both HF, patients with fever or with malaria symptoms were tested at the place where the COVID-19 screening was conducted, and medication was also prescribed when the RDT was positive for malaria, thus the access of patients with feverish syndrome was limited at the HF. At the CHW It was found that, from January to May 2020, there was a decrease in people seeking medical care and malaria cases (unrecorded count).

### Malaria epidemiological data analysis

**Figure 1:**
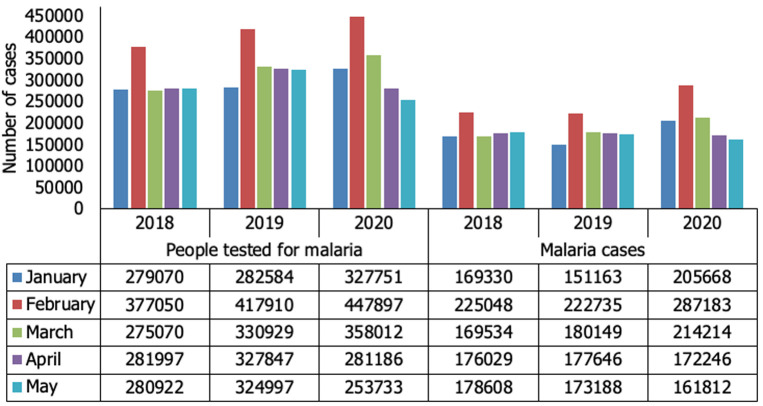
distribution of people tested for malaria, and malaria cases in health facilities from January to May, 2018 to 2020, Nampula Province

**Figure 2:**
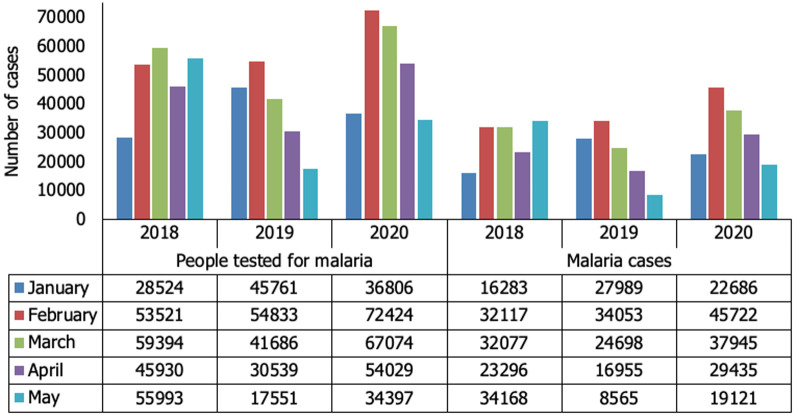
distribution of people tested for malaria, and malaria cases in community health worker from January to May, 2018 to 2020, Nampula Province

The number of patients tested for malaria in HF, there was an overall decrease of 1.0% (1.668.579/1.684.267) from 2019 to 2020, and on COVID-19 emergency period, the decrease was 14.0% (281.186/327.847) comparing April 2019 to 2020, and 22.0% (253.733/324.997) comparing May of the same period (Figure 1). The malaria cases, there was a general increase of 15.0% (904.881/1.041.123) from 2019 to 2020 however, comparing cases in April 2019 to 2020, there was a 3.0% (177.646/ 172.246) decrease, and in May a 7.0% (173.188/161.812) decrease of malaria cases (Figure 2). In CHW, there was an increase in the number of people tested for malaria by 39.0% (190.370/264.730) from 2019 to 2020, and the same trend occurred to the malaria cases, with an increase of 38.0% (112.260/154.909). On COVID-19 pandemic emergency, there was an increase in the number of malaria cases, of 53.6% (24.698/37.945) in March, 73.6% (16.955/29.435) in April, and more than 100.0% (8.565/19.121) in May (Figure 2).

## Discussion

The higher consumption of AL may be associated with the fact that it is the first choice in the first line of malaria treatment. No matter how little the stock is, other drugs from the same treatment line, such as artesunate-amodiaquine (ASAQ) and oral quinine, can be used [6]. In Mozambique there was an increase in the number of malaria cases diagnosed by the CHW, compared to HF, and to reinforce the diagnosis and treatment more RDT and artemisinin-combination therapies were distributed to CHW instead of the HF [7,8].

The integration of malaria testing at COVID-19 screening sites allowed malaria surveillance to be unaffected in Mozambique. The confirmation of a malaria infection does not rule out the possibility that the patient might also be suffering from COVID-19, similarly, suspected or confirmed COVID-19 patients in malaria-endemic areas should also be tested for malaria [9]. The interruption of malaria surveillance services due to COVID-19 could result in an increase of over 20% in malaria cases and double malaria mortality in Africa, as happened in the Democratic Republic of Congo, were due to the increased attention to Ebola, there were more deaths from malaria [5,10-12].

The increase of malaria tested people and cases between January and February in the year 2020 followed the trend of the years 2018 and 2019, and it's in this period that the peak rainfall at the country occurs.

Even with the same trend, the higher number of people tested for malaria and malaria cases registered at community, compared to HF, may be the result of people not going to the HF due to fear of contracting COVID-19, and the media´s “stay home” as one of the ways to reduce the risk of contracting COVID-19. The susceptibility of adults and the elderly to COVID-19, fear of being contaminated can make them reluctant to join HF [11,13]. An unpublished mortality assessment carried out in Nampula Province under COVID-19 surveillance found that in the civil registry and notary service the majority cause of death in the first half of 2020 was malaria, with an increase of over 100% compared to the same period of the year 2019. The decrease in people tested for malaria in HF during the COVID-19 emergency period may cause an increase in the number of people with parasite load and deaths in the community, and it can also increase the prevalence of malaria, and resistance to malaria drugs, as people may choose to self-medicate rather than go to HF.

## Conclusion

The stock of antimalarial medicines may have been sufficient to meet patient demand in the HF, as the number of cases of malaria registered in the HF has been decreased. The declaration of a state of emergency may have influenced the decrease in the number of people tested for malaria in HF, and this may have contributed to the overload of activities for CHW.

### What is known about this topic

- The COVID-19 pandemic brought new challenges in the continuity of malaria diagnosis and treatment;
- In malaria-endemic countries, the disruption of malaria control interventions due to COVID-19 could increase malaria prevalence and mortality.

### What this study adds

- With the decrease of patients in the HF during the COVID-19 emergency, there was an increase in demand for CHW, and the HF had to be restructured to ensure continuity of the services;
- The CHW had an important role in malaria surveillance during the COVID-19 pandemic period.
